# The Medical Treatment of Women in India

**Published:** 1887-07-16

**Authors:** 


### The Medical Treatment of Women in India.

By a Supporter of the Countess of Dufferin's Fund.

"Brahmin women permit male medical attendance, and there are only a few castes now which will not allow a medical man to attend them in confinements, and the opposition of these is slowly giving way," etc.

The above statement in a letter, signed "H. F. B., Nasik, Bombay," which appeared in *The Queen* of the 12th March, was so at variance with the usually received opinion of the subject, that a Hindu gentleman of position was asked how far he considered the statement correct; and as his reply is that of one who writes with authority, I beg to give it in full:—

"The statement you refer to above is certainly, at first sight, a mischievous one, and it becomes more so as there is just a grain of truth in it to give it weight in the eyes of those who, in England, cannot embarrass themselves with any careful examination of facts.

"From my experience of Eastern India I cannot deny that male medical attendance is sometimes permitted by Hindus in difficult delivery cases. The same may be said of other cases too, and to a larger extent. *Kavirayes* and *Vaids* in Hindu houses, and *Hakims* in Mahomedan houses, are frequently consulted for the relief of Indian ladies. People in the higher and middle ranks of life in Bengal do also, to a certain extent, avail themselves of the services of male practitioners, trained in European medical science, for the treatment of their female relations. In Calcutta, Benares, and elsewhere, European physicians are likewise occasionally called in for the treatment of serious cases. Matters could not be otherwise; when life is in danger, and relief, real or pretended, is at hand, human nature must prevail, and local, tribal or racial prejudices must go to the wall. But these facts do not at all cover the ground before us. As far as I am aware, it has not been urged by anyone that the whole of womankind in India obtained no medical relief whatever, and that male practitioners are absolutely prohibited from treating females. What is complained of is not *total* want, but an extent of insufficiency amounting to it, for general purposes.

"To show this clearly, I must enter into a few details. In the first place, there are various female complaints which, owing to local causes, are perhaps more prevalent in India than in Europe, and for them there is no adequate medical aid available. Unwilling, from a sense of delicacy, to communicate even to their male relatives the character and symptoms of their complaints, native ladies either directly consult or, through their maid-servants, obtain whatever help they can get from village midwives and quacks of their own sex, and the result generally is very serious. When the disease becomes dangerous, competent physicians are sometimes, but not often, called in; but they come too late, and, generally speaking, Indian ladies have to depend, for all practical purposes, on the curative powers of nature.

"Such is likewise the case as regards medical and surgical aid in the lying-in chamber. Our *Vaids* and *Hakims* do not study the character of puerperal diseases, nor as a rule undertake to attend to them, and the whole duty of puerperal management devolves on our midwives, who, as a body, are utterly incompetent. They belong to the lowest grade of society, and are never trained to their work. When widowed and old, women of the lowest caste, such as *Domes*, *Chamars*, and *Podes*, etc., first seek employment as attendants on women in confinement, and after a time set themselves up as midwives. There is thus no help for Indian women at the most critical period of their lives, except what may be obtained from these so-called *Dhaies*.

"Of late the Calcutta Medical College has trained some midwives, but their number is extremely limited, and only a few have gone to the country districts. Their total number cannot be reckoned by scores. The Government Medical Schools do not as a rule teach midwifery, and but few assistant-surgeons take up that branch of their profession; consequently, the distribution of means for the treatment of puerperal cases is as extreme as possible, and hence the high rate of mortality during childbirth among the native population.

"For the treatment of diseases common to both sexes the means available is certainly much better, but under the peculiar circumstances of the country it cannot be utilised to any large extent; for the higher, the middle, and even the lower-middle classes of Mahomedans, do not as a rule allow male practitioners to enter their zenana, and the treatment of females has to be carried on under exceedingly disadvantageous circumstances Symptoms are the only guides to diagnosis, and in a Mahomedan house the physician cannot himself study the symptoms of the disease he has to treat. He cannot be permitted to behold the appearance of his patient, or even to feel her pulse. He has to depend all but entirely upon the information communicated to him by the stupid maid-servants, whose language and ideas are generally anything but precise; and treatment under the circumstances cannot in the nature of things be at all satisfactory.

"In Hindu houses permission is always granted to feel the pulse, but in such cases the patient remains enveloped in a thick sheet or quilt, and only thrusts out her left hand as far as the wrist, and the physician can know nothing of her appearance. The face is rarely exposed, and when it is the eyes are kept firmly closed. Out of Calcutta the tongue is seldom, if ever, shown to the physician, and even in Calcutta it is shown with very great reluctance. The heat of the skin may be judged by touching the forehead, but no other tactual examination is permitted except from above a thick sheet or quilt, and that in not ordinary cases.

"Under these circumstances it is no exaggeration to say that Indian women of the respectable or genteel classes derive very little professional aid from qualified physicians. What they do derive I have detailed above, and that is as often beneficial as hurtful. The ablest physicians can do but little good if they are not allowed the fullest scope for the exercise of their profession, and in the case of Indian ladies that scope is often so circumscribed as to amount to a negative. It is undeniable that Brahmin and other high-caste ladies are at times subjected to surgical operations by European doctors, but that is quite exceptional, and does not in the least affect the real question at issue. That question is the present state of medical aid for women in India, and none can gainsay the fact that it is extremely deficient. Duly qualified medical practitioners are not yet numerous in the country, and whatever their number, they, being of the male sex, are not generally called in for the treatment of females, as I have stated; and as it is not easy readily to overcome the prejudices of a nation of over two hundred millions, if help has to be rendered it must be through the medium of a duly qualified band of female practitioners."

The subject is one which cannot fail to interest those who have contributed, or who desire to contribute, to the support of the National Association for Supplying Female Medical Aid to the Women of India, and is of great importance to those who are helping the Countess of Dufferin in her efforts to alleviate the sufferings of our Indian sisters. All contributions should be paid direct to Messrs. Coutts & Co., 59, Strand, W.C. All correspondence should be addressed to the Honorary Secretary, the Countess of Dufferin's Fund, Viceroy's Camp, India.

